# Berberine improved experimental chronic colitis by regulating interferon-γ- and IL-17A-producing lamina propria CD4^+^ T cells through AMPK activation

**DOI:** 10.1038/s41598-019-48331-w

**Published:** 2019-08-15

**Authors:** Masahiro Takahara, Akinobu Takaki, Sakiko Hiraoka, Takuya Adachi, Yasuyuki Shimomura, Hiroshi Matsushita, Tien Thi Thuy Nguyen, Kazuko Koike, Airi Ikeda, Shiho Takashima, Yasushi Yamasaki, Toshihiro Inokuchi, Hideaki Kinugasa, Yusaku Sugihara, Keita Harada, Shingo Eikawa, Hidetoshi Morita, Heiichiro Udono, Hiroyuki Okada

**Affiliations:** 1 Department of Gastroenterology and Hepatology, Okayama University Graduate School of Medicine, Dentistry and Pharmaceutical Sciences, 2-5-1 Shikata-cho, Kita-ku, Okayama 700-8558 Japan; 2 Department of Animal Applied Microbiology, Okayama University Graduate School of Environmental and Life Science, 1-1-1 Tsushima-naka, Kita-ku, Okayama 700-8530 Japan; 3 College of Agriculture and Forestry, Hue University, 3 Le Loi, Hue City, Vietnam; 4 Department of Immunology, Okayama University Graduate School of Medicine, Dentistry and Pharmaceutical Sciences, 2-5-1 Shikata-cho, Kita-ku, Okayama 700-8558 Japan

**Keywords:** T-helper 1 cells, Mucosal immunology

## Abstract

The herbal medicine berberine (BBR) has been recently shown to be an AMP-activated protein kinase (AMPK) productive activator with various properties that induce anti-inflammatory responses. We investigated the effects of BBR on the mechanisms of mucosal CD4^+^T cell activation *in vitro* and on the inflammatory responses in T cell transfer mouse models of inflammatory bowel disease (IBD). We examined the favorable effects of BBR *in vitro*, using lamina propria (LP) CD4^+^ T cells in T cell transfer IBD models in which SCID mice had been injected with CD4^+^CD45RB^high^ T cells. BBR suppressed the frequency of IFN-γ- and Il-17A-producing LP CD4^+^ T cells. This effect was found to be regulated by AMPK activation possibly induced by oxidative phosphorylation inhibition. We then examined the effects of BBR on the same IBD models *in vivo*. BBR-fed mice showed AMPK activation in the LPCD4^+^ T cells and an improvement of colitis. Our study newly showed that the BBR-induced AMPK activation of mucosal CD4^+^ T cells resulted in an improvement of IBD and underscored the importance of AMPK activity in colonic inflammation.

## Introduction

Inflammatory bowel disease (IBD) is characterized by chronic inflammation of the gastrointestinal tract, occurring primarily in young individuals.

Accumulating evidence suggests that IBD is caused by an inappropriate response of the innate and acquired immune systems to the commensal microbiota^1^. Among these immune systems, inflammatory CD4^+^ T cells in the colonic lamina propria (LP) are acknowledged as critical factors in the pathogenesis of IBD^2^.

Berberine (BBR) is a traditional Chinese herbal medicine extracted from Phellodendron bark and Coptis japonica and is used to treat gastrointestinal disorders, such as diarrhea, with few adverse events. BBR also exerts various types of effects, such as anti-tumor, anti-diabetic and anti-inflammatory effects^3–8^. BBR is known to activate AMPK, which is an enzyme that plays a role in cellular energy homeostasis and different fundamental cellular processes, including the cell proliferation, survival and metabolism. The beneficial effect of BBR has recently been considered to be due to the activation of AMPK^9,10^. The energy metabolism changes in immune cells have been accepted to be involved in immune regulation. Furthermore, AMPK signaling has been shown to inhibit the inflammatory responses^11–16^. Therefore, BBR may be of potential therapeutic utility in the treatment of immune-mediated diseases.

Several reports have already shown the utility of BBR in the treatment of IBD^17–20^. Various mechanisms, such as repair of the epithelial barrier function and the regulation of innate and adoptive immune responses, have been indicated in these reports. However, detailed analyses, including the mechanisms of the AMPK activity against CD4^+^ T cells, especially LP CD4^+^ T cells, which are considered to be involved in the pathogenesis of IBD^2^, have not been performed. In addition, the widely used models of colitis are chemically induced, which is not appropriate for representing the chronic inflammatory features of IBD.

In this report, we investigated the effect and inhibitory mechanisms of BBR on LP CD4^+^ T cells of IBD—in terms of the cellular energy metabolism related to AMPK activity—using a T cell transfer colitis model involving the transfer of naïve (CD4^+^CD45RB^high^) T cells into congenic immunodeficiency mice to induce CD4^+^ T cell-specific colitis. Our results provide new insight into how BBR regulates the LP CD4^+^ T cell function as well as underscore the importance of AMPK activity in colonic inflammation. In the future, given these results, the involvement of AMPK activity in colonic inflammation may lead to the development of new therapeutic targets that are safe and useful for treating IBD.

## Results

### BBR suppressed inflammatory cytokines of LP CD4^+^ T cells from colitis SCID mice *in vitro*

**Figure 1:**
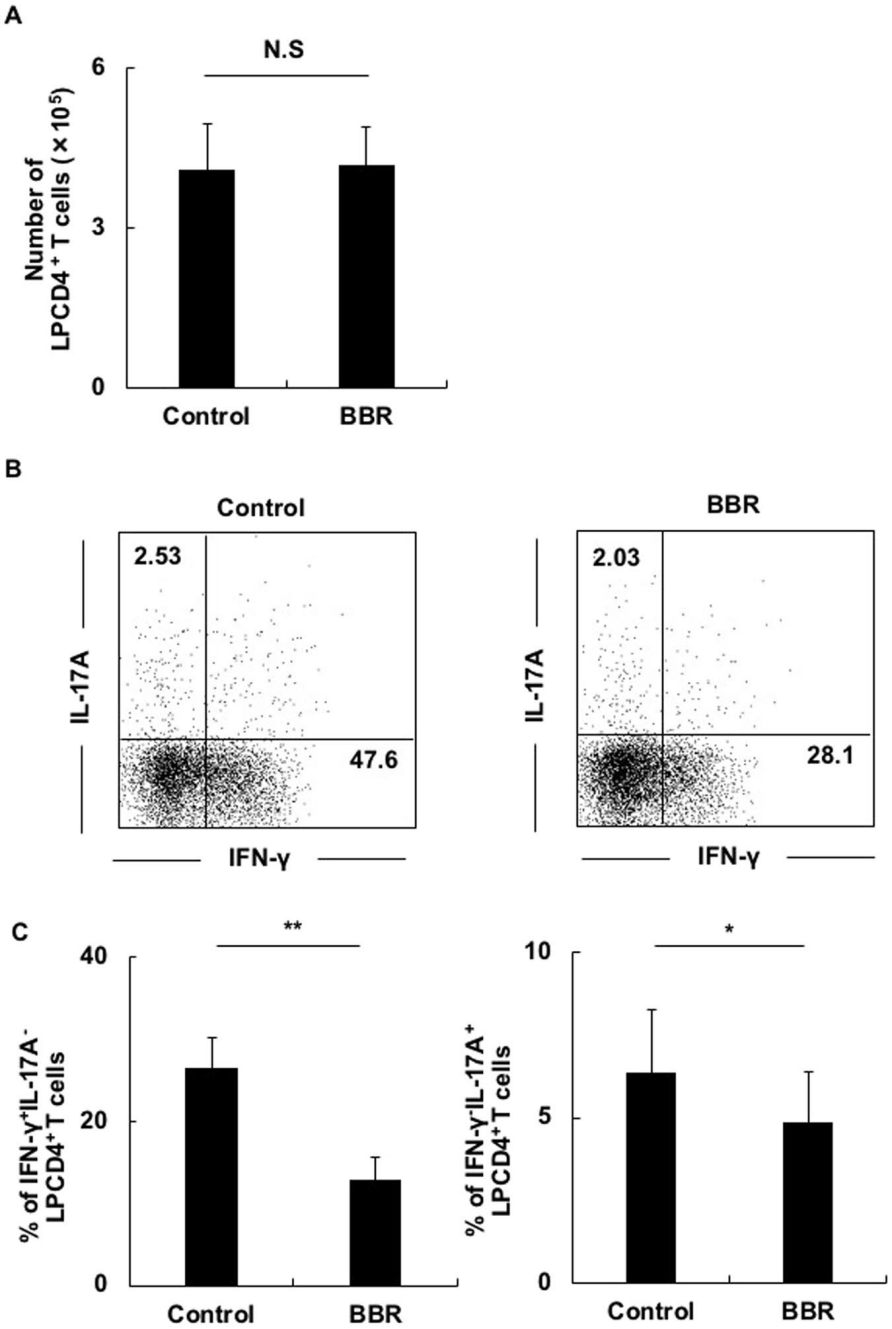
BBR suppressed inflammatory cytokines of LP CD4^+^ T cells from colitis SCID mice *in vitro*. (**A**) The cytotoxicity analysis of BBR. Colitis LP CD4^+^ T cells were stimulated with PMA plus ionomycin mixed with BBR (BBR) or not (Control). The bar graphs show number of LP CD4^+^ T cells. (**B**) Colitis LP CD4^+^ T cells were stimulated with PMA plus ionomycin mixed with BBR (BBR) or not (Control) for 8 h. After that, cells were collected, and intracellular staining was performed to analyze the CD3^+^CD4^+^IFN-γ^+^- or IL-17A^+^-producing cells by flow cytometry. Representative flow cytometry images are shown. Flow cytometry showed the percentage of CD3^+^CD4^+^IFN-γ^+^-or IL-17A^+^-producing cells in colitis LP CD4^+^ T cells. (**C**) The bar graphs show the percentage of IFN-γ- and IL-17A-producing cells in LP CD4^+^ T cells. All data are reported as the mean ± SEM. N is 6 in each group. N.S. not significant. *P < 0.05, **P < 0.01.

We first assessed the inhibitory effect of BBR on LP CD4^+^ T cell activation *in vitro*. LP CD4^+^ T cells of colitis SCID mice (colitis LP CD4^+^ T cells) injected with CD4^+^ CD45RB^high^ T cells were isolated and stimulated with PMA plus ionomycin mixed with BBR or not. In this colitis model, a large amount of the Th1-related cytokine IFN-γ and a small amount of the Th17-related cytokine IL-17A are known to be secreted by LP CD4^+^ T cells^21,22^. Therefore, we analyzed the production of IFN-γ and IL-17A. BBR exerted a dose-dependent inhibitory effect but showed no cytotoxic effect at concentrations of 100 µM (Fig. 1A, Supplementary Fig. S1). Therefore, we performed subsequent experiments with BBR 100 µM.

As shown in Fig. 1B,C, the frequency of IFN-γ-producing CD4^+^ T cells in BBR-treated colitis LP CD4^+^ T cells was significantly lower than in non-BBR-treated colitis LP CD4^+^ T cells. The frequency of IL-17A-producing CD4^+^ T cells was also lower in BBR-treated cells than in untreated cells. These results indicated that BBR directly affected the colitis LP CD4^+^ T cells, thereby reducing the Th1/Th17 responses.

### BBR suppressed the Th1/Th17-related JAK/STAT pathway of LP CD4^+^ T cells collected from colitis SCID mice

**Figure 2:**
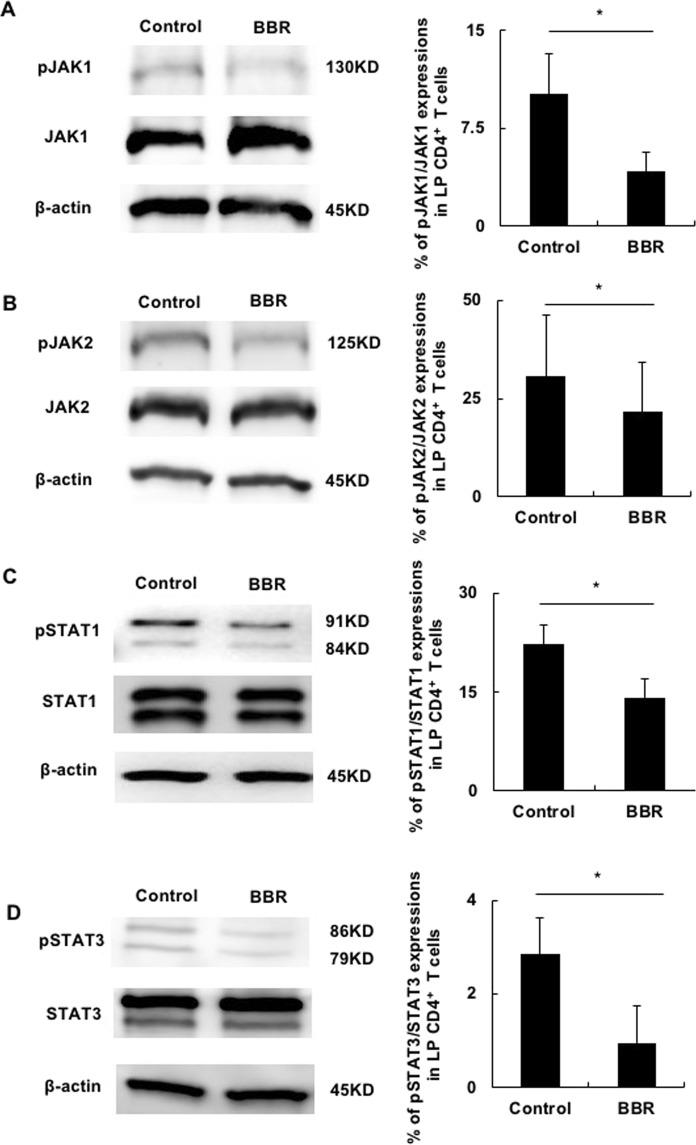
BBR suppressed the Th1/Th17-related JAK/STAT pathway of LP CD4^+^ T cells collected from colitis SCID mice. (**A**–**D**) Colitis LP CD4^+^ T cells stimulated with PMA plus ionomycin were collected and analyzed for the JAK/STAT pathway by Western blotting.The left figures show the representative Western blotting images of each protein. The cropped blots are used in the figure, and full-length blots are presented in Supplementary Fig. S2. All gels were run in the same experimental conditions (see material and methods for details). The bar graphs show the percentage of each protein expression. All data are reported as the mean ± SEM. N is 5 in each group. *P < 0.05.

We further investigated the expression of signal molecules correlated with Th1/Th17 activation. The JAK/STAT pathway is a major signal that regulates Th cell differentiation and their function^23,24^. The Th1-related signal transduction molecules JAK1, JAK2, and STAT1 were all suppressed by BBR addition. The Th17-related molecule STAT3 was also suppressed by BBR addition (Fig. 2A–D). These results further confirmed the direct effect of BBR on the immune responses in colitis LP CD4^+^ T cells.

### BBR increased the AMPK activity and regulated the IFN-γ and IL-17A secretion from colitis LP CD4^+^ T cells

**Figure 3:**
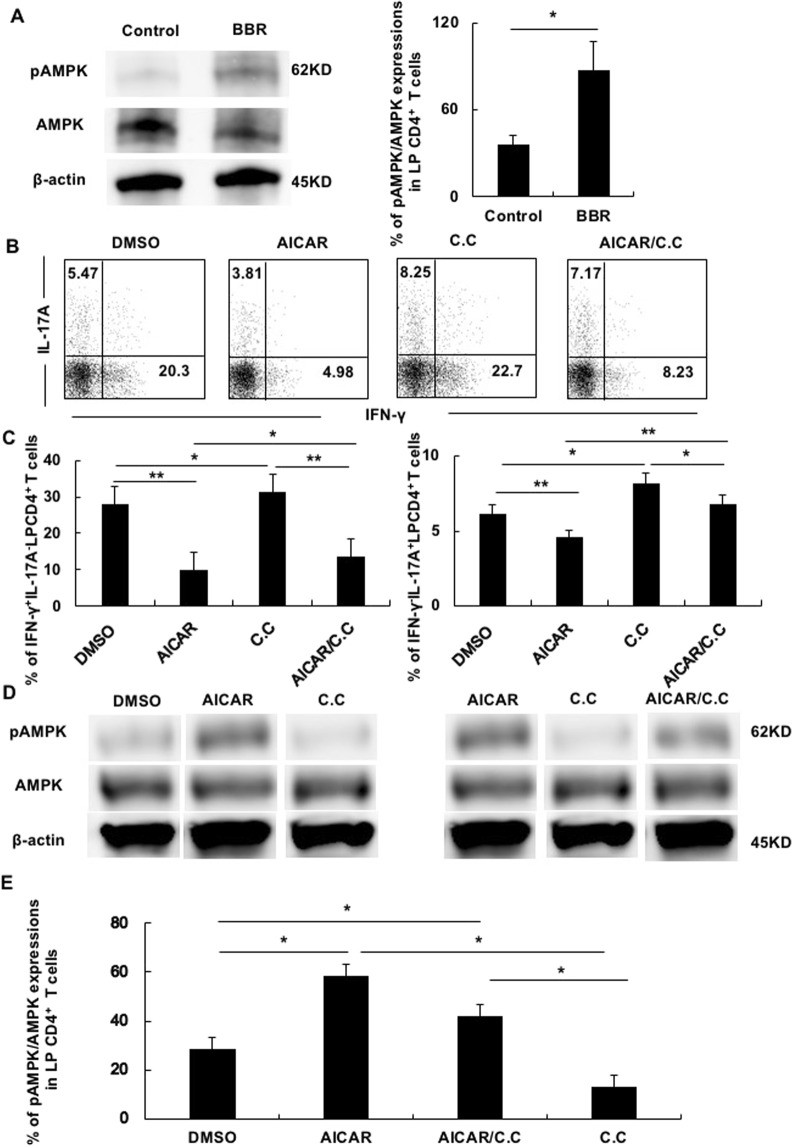
BBR increased AMPK activity and regulated the IFN-γ and IL-17A secretion from colitis LP CD4^+^ T cells. (**A**) The phosphorylation of AMPK at Thr172 levels in colitis LP CD4^+^ T cells stimulated with PMA plus ionomycin mixed with BBR (BBR) or not (Control) was detected by Western blotting. The left figure is a representative blotting image. The right figure is the percentage of pAMPK/AMPK expression. (B-E) Colitis LP CD4^+^ T cells were treated with DMSO, AICAR (250 μM), AICAR (250 µm) mixed with C.C. (400 nM) or C.C. alone (400 nΜ) for 30 minutes and stimulated with PMA plus ionomycin for 4 h. After stimulation, cells were collected, and intracellular staining was performed to analyze the CD3^+^CD4^+^IFN-γ^+^- or CD3^+^CD4^+^IL17A^+^-producing cells by flow cytometry. The cells were then analyzed for AMPK at Thr172 levels by Western blotting. (**B**) The figure shows the representative flow cytometry. (**C**) The bar graphs show the percentage of cytokine-producing cells. (**D**) The figure shows the representative Western blotting images of AMPK at Thr172 levels. (**E**) The bar graphs show the percentage of pAMPK/AMPK expression. The cropped blots are used in the figure, and full-length blots are presented in Supplementary Fig. S3. All gels were run in the same experimental conditions (see material and methods for details). All data are reported as the mean ± SEM. N is 5 in each group. *P < 0.05, **P < 0.01.

Given that recent reports indicated that AMPK plays a key role in T cell activation and that BBR increased AMPK activity, we hypothesized AMPK activity as a vital mechanism on BBR effects. Earlier studies had established that phosphorylation of AMPK at Thr172 correlates with AMPK activity^25^. We therefore first compared the AMPK expression by Western blotting between BBR-treated colitis LP CD4^+^ T cells and non-BBR-treated colitis LP CD4^+^ T cells. As expected, the phosphorylation state of AMPK at Thr172 (p-AMPK) expressions was significantly increased in BBR-treated colitis LP CD4^+^ T cells (Fig. 3A).

Next, we examined the relationship between the AMPK activity and inflammatory cytokines. We measured IFN-γ- and IL-17A-producing colitis LP CD4^+^ T cells using AMPK agonist, 5-Aminoimidazole-4-carboxamide ribonucleotide (AICAR) and antagonist, Compound C (C.C.). The addition of AICAR resulted in the suppression of the frequency of IFN-γ- and IL17A-producing colitis LP CD4^+^ T cells. In contrast to the effect of AICAR on colitis LP CD4^+^ T cells, the addition of C.C. increased the frequency of IFN-γ-producing colitis LP CD4^+^ T cells as well as that of IL-17A-producing LP CD4^+^ T cells (Fig. 3B,C). Furthermore, the addition of mixing with AICAR and C.C. increased the frequency of IFN-γ- and IL-17A-producing colitis LP CD4^+^ T cells in comparison to the addition of AICAR alone and decreased the frequency of IFN-γ- and IL-17A-producing colitis LP CD4^+^ T cells in comparison to the addition of C.C. alone (Fig. 3B,C).

Western blotting analysis showed that the p-AMPK activity of colitis LP CD4^+^ T cells was significantly increased by AICAR treatment and significantly suppressed by C.C. treatment (Fig. 3D,E). Furthermore, the p-AMPK activity of colitis LP CD4^+^ T cells was significantly suppressed by combination AICAR and C.C. treatment in comparison to AICAR and increased in comparison to C.C. (Fig. 3D,E).

These data strongly indicated that the production of IFN-γ and IL-17A by colitis LP CD4^+^ T cells was regulated through AMPK activities.

### BBR affected the oxidative phosphorylation and decreased the total adenosine triphosphate production

**Figure 4:**
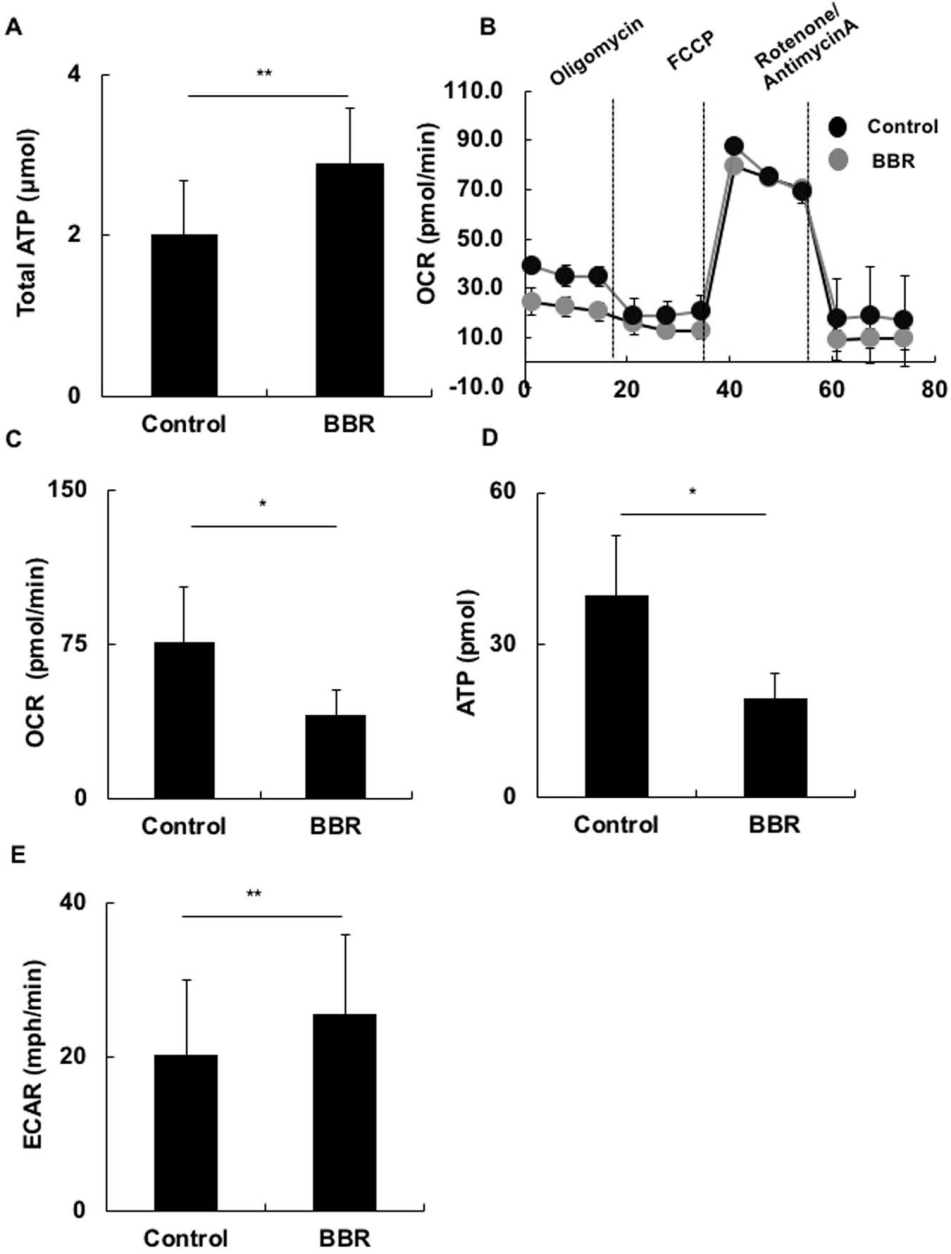
BBR affected the oxidative phosphorylation and decreased the total adenosine triphosphate production. Colitis LP CD4^+^ T cells were stimulated with PMA plus ionomycin mixed with BBR (BBR) or not (Control) for 8 h. (**A**) After culture, the cells were collected, and the intracellular ATP was analyzed. (**B**) Bioenergetic profile of colitis LP CD4^+^ T cells. The OCR profile following the addition of mitochondrial inhibitors (oligomycin, FCCP, rotenone/antimycin A) was determined by a Flux analyzer xXF 96 S. (**C**–**E**) The effects of BBR on the OCR, mitochondrial ATP and ECAR profiles were separately analyzed. All data are reported as the mean ± SEM. N is 5 in each group. *P < 0.05, **P < 0.01.

AMPK is activated in response to stresses that deplete cellular adenosine triphosphate (ATP), such as low glucose, hypoxia, ischemia and heat shock^26–28^. Therefore, we compared the ATP production in BBR-treated colitis LP CD4^+^ T cells to that of non-BBR-treated colitis LP CD4^+^ T cells. As expected, the ATP production of BBR-treated colitis LP CD4^+^ T cells was significantly decreased relative to that of non-BBR-treated colitis LP CD4^+^ T cells (Fig. 4A).

ATP is produced mainly by two routes, glycolysis and oxidative phosphorylation (OXPHOS). Glycolysis is the metabolic pathway that extracts energy from glucose, which is used to reform ATP. OXPHOS is the metabolic pathway by which cells use enzymes to oxidize nutrients (glucose, fat, protein), thereby releasing energy, which is used to reform ATP. This takes place inside mitochondria. We therefore examined whether or not BBR worked on these two routes. We measured the glycolysis marker extracellular acidification rate (ECAR) and the OXPHOS marker oxygen consumption rate (OCR). BBR-treated colitis LP CD4^+^ T cells showed suppressed OCR and ATP productions (Fig. 4B–D). In contrast, BBR-treated colitis LP CD4^+^ T cells showed elevated ECAR (Fig. 4E). This was due to the effect of increased AMPK activation compensating for the loss of ATP production in OXPHOS. However, according to the total ATP production in our experiment, glycolysis did not sufficiently compensate for ATP production.

These experiments suggest that BBR suppressed OXPHOS and total ATP production, resulting in AMPK activation.

### BBR ameliorated experimental colitis *in vivo* with changes in gut microbiota and decreased the production of IFN-γ and IL-17A

**Figure 5:**
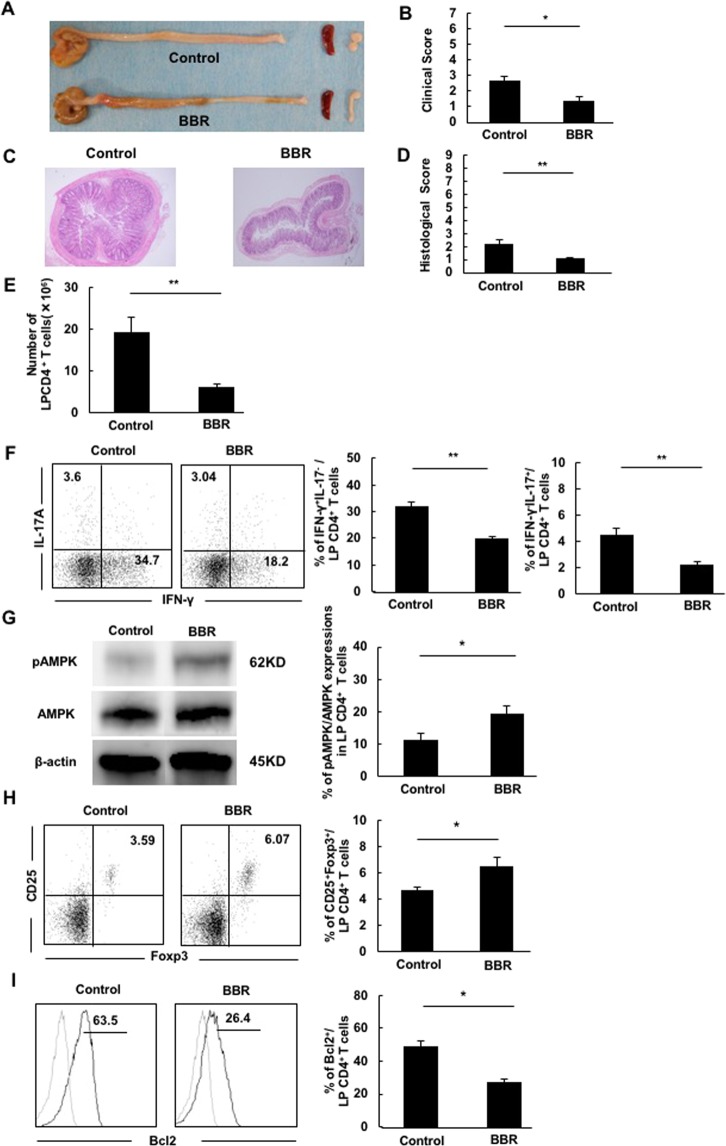
BBR ameliorated experimental colitis in vivo with changes in gut microbiota and decreased the production of IFN-γ and IL-17A. A total of 3 × 10^5^ CD^4+^CD45RB^high^ T cells of Balb/c mice were transferred into new SCID mice to establish a colitis model (n = 6 per group). After that, a diet including BBR (BBR) or not (Control) was given to each group. The mice were monitored for up to seven weeks and then sacrificed and analyzed. (**A**) The gross appearance of the colon, spleen and mesenteric lymph nodes. (**B**) Clinical scores. (**C**) Histopathology of the distal colon. Original magnification: ×40. (**D**) Histological scores. Pictures show representative samples from each group. (**E**) Number of LP CD4^+^CD3^+^ T cells. (**F**) Intracellular staining of IFN-γ- and IL-17A-producing cells in LP CD4^+^ T cells. Flow cytometry and a bar graph showing the percentage of CD3^+^CD4^+^IFN-γ^+^- or IL-17A^+^-producing cells in colitis LP CD4^+^ T cells. (**G**) The AMPK expression of LP CD4^+^ T cells in both groups. The bar graph shows the percentage of pAMPK/AMPK expression. (**H**) The expression of CD25^+^Foxp3^+^ Treg on CD3^+^CD4^+^ T cells in the LP. (**I**) The expression of Bcl2^+^ on CD3^+^CD4^+^ T cells in the LP. Cropped blots are used in the figure; full-length blots are presented in Supplementary Fig. S5. All gels were run in the same experimental conditions (see Materials and methods for details). The numerical values represent the mean values of 6 samples per group. Pictures and dot plots of flow cytometry show representative samples from each group. All data are shown as the mean ± SEM for 6 mice per group. *P < 0.05, **P < 0.01.

Next, we assessed the BBR effects on SCID colitis mouse model which injected with CD4^+^CD45RB^high^ T cells. We gave colitis mice a diet mixed with BBR or not and evaluated the severity of colitis. BBR at a concentration of 0.12% was not effective (Supplementary Fig. S4), but that at 0.35% was effective. The clinical and histological scores of the BBR diet group were lower than those of the non-BBR diet group (Fig. 5A–D).

To further confirm the effect of BBR on experimental colitis, changes in the immune reactions were examined. The numbers of CD4^+^ T cells in LP were reduced by BBR administration (Fig. 5E). The numbers of IFN-γ- or IL-17A-producing LP CD4^+^ T cells were lower in the BBR diet group than in the non-BBR diet group (Fig. 5F). We evaluated the AMPK expression of LP CD4^+^ T cells in both groups; the expression in the BBR-treated group was significantly higher than in the non-BBR-treated group (Fig. 5G).

Given that BBR has been shown to regulate Treg cells by modifying the microbiota in a chemically induced IBD model and to regulate Bcl-2 in an *in vitro* autoimmune disease model^29,30^, we next examined the change in the frequency of Foxp3^+^ Treg cells and the Bcl-2 expression. The frequency of Foxp3^+^Treg of LP CD4^+^ T cells were higher and Bcl-2^+^ of LP CD4^+^ T cells were lower in the BBR diet group than in the non-BBR diet group (Fig. 5H,I).

These data indicated that BBR reduced the T cell inflammatory responses and ameliorated experimental colitis.

**Figure 6:**
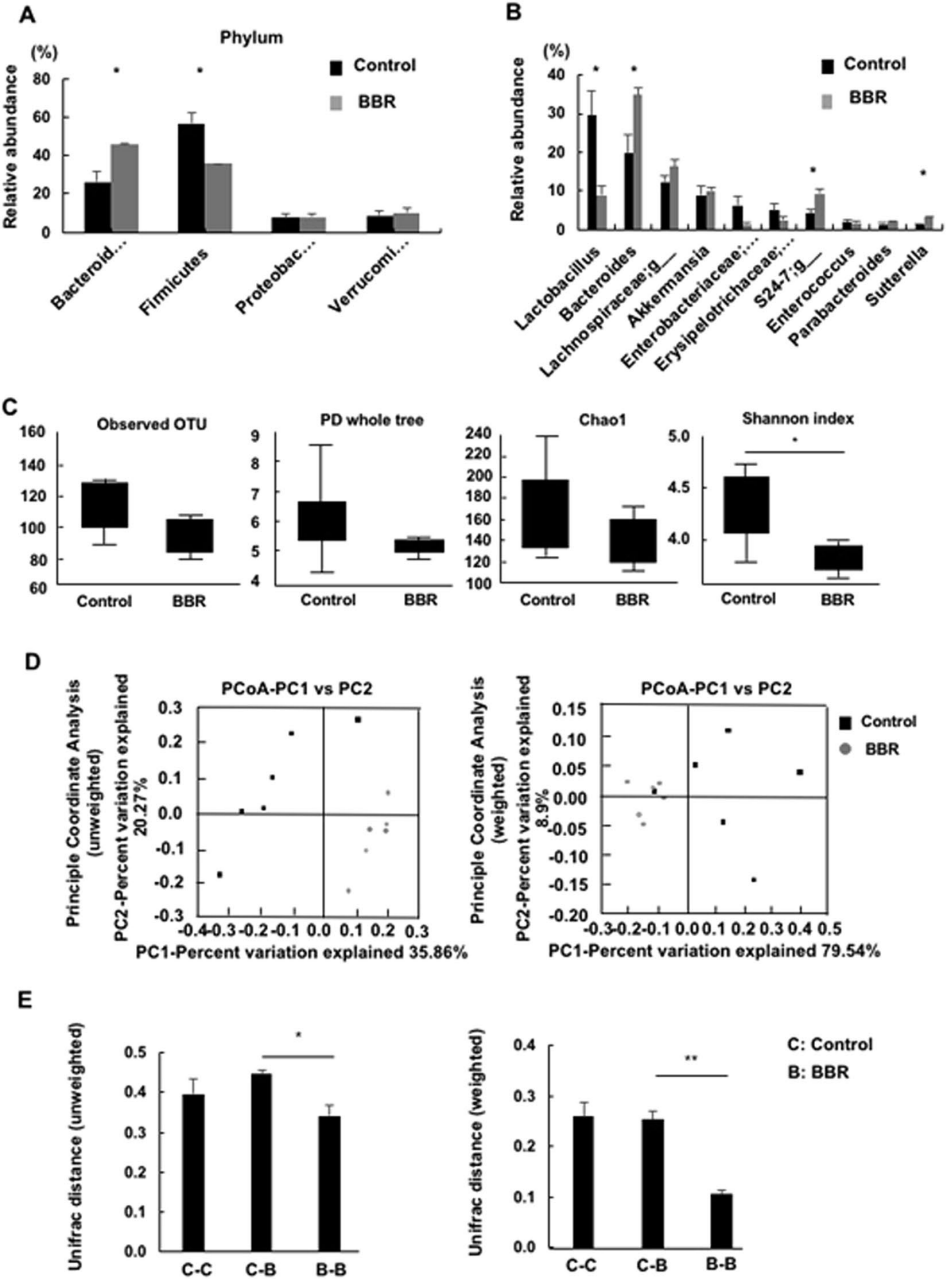
(**A**,**B**) A comparison of the fecal microbial community at the phylum and genus level between colitis and BBR. (**C**) Alpha diversity_Observed OTU, PD whole tree, Chao1 and Shannon index. (**D**,**E**) Beta diversity_UniFrac_unweighted and UniFrac unweighted distance. Each point represents the gut microbiota community of each mouse, with black points and gray points indicating the colitis and BBR groups, respectively. All data are shown as the mean ± SEM for 6 mice per group. *P < 0.05, **P < 0.01.

Gut microbiota disorder is a known pathogenesis of IBD^1^. Therefore, we also examined the gut microbiota using fecal samples of colitis mice at the time of sacrifice. In both groups, the gut community was mainly built by four major phyla, and significant differences were noted in the rates of the *Bacteroides* and Firmicutes phyla (Fig. 6A). Regarding lower classifications, genus-level comparisons showed statistically significant dissimilarities in *Lactobacillus*, *Bacteroides*, unidentified genus of S24-7 family and *Sutterella* found in the top 10 most abundant genera of both groups (Fig. 6B).

The alpha diversity was evaluated to assess the species richness within the gut microbiota community diversity of the two groups (Fig. 6C). There were no considerable differences in the number of observed OTU, Faith’s phylogenetic diversity whole tree and the diversity of the species estimated by Chao1. However, interestingly, the species richness and evenness calculated by the Shannon index were significantly reduced with BBR supplementation.

The gut microbiota community was measured not only based on the diversity within each group but also between the two groups to provide an overview of the gut microbiota structure. An unweighted and weighted UniFrac Principle Coordinate Analysis (PCoA) and the UniFrac distance of gut microbiota community in both groups were analyzed (Fig. 6D). Both scatter plots demonstrated that gut microbiota structure was changed in response to BBR administration. The gray dots representing the BBR diet group gathered closely, while the black dots representing the non-BBR diet group seemed scattered. The largest principle coordinates (PC1) were quite high, accounting for 35.86% and 79.73% of the total variation in the unweighted and weighted analyses, respectively. In addition, 20.27% and 8.99% of the total variation were the values of second principle coordinate (PC2), corresponding to the unweighted and weighted analyses. The shifting of the gut composition under BBR intake was indicated in bar plots of the unweighted and weighted UniFrac distance analyses (Fig. 6E). These data suggested that BBR affected the gut microbiota composition, and this change might be one of the factors ameliorating colitis.

## Discussion

In this report, we showed that BBR ameliorated CD4^+^ T cell-related chronic colitis in a mouse model and revealed the relationship between BBR and AMPK, including its metabolic pathway, through *in vitro* and *in vivo* experiments.

Our message based on the present data can be summarized in the following three points: 1) We adapted the CD4^+^ T cell-transfer model, a chronic colitis model that clearly differs from the widely used chemically induced acute colitis models; 2) BBR affected the AMPK-related metabolic pathway and reduced the inflammatory responses; and 3) BBR reduced the microbiota diversity, possibly by its antimicrobial action.

We used CD4^+^T cell-transfer colitis models in our *in vitro* and *in vivo* experiments, although most previous reports used colitis models in which colitis was induced by chemicals, such as DSS^17–20^. Such chemically induced models showed acute colitis that did not accurately reflect the chronic inflammatory process observed in IBD patients. CD4^+^T cell-transfer colitis models show chronic colitis with T cell infiltration, dependent on the microbiota, and may be superior to chemically induced models. The use of CD4^+^T cell-transfer colitis models revealed a new inhibitory mechanism of BBR, particularly in relation to colitis LP CD4^+^ T cells.

The beneficial effects of BBR have been considered to occur due to the activation of AMPK, an energy metabolic sensor crucial for cellular energy homeostasis^9,10^. Recently, many reports have revealed that the immune metabolism is a key factor for controlling the immune cell function^11–16,31,32^. We therefore focused on AMPK and attempted to reveal its immunological effects, particularly in relation to colitis LP CD4^+^ T cells, data on which are scarce. Our results showed that BBR increased the AMPK activity in colitic LP CD4^+^ T cells. In addition, we showed that the AMPK activity regulated IFN-γ- and IL-17A-producing LP CD4^+^ T cells. These data suggested that the regulation of AMPK in colitis LPCD4^+^ T cells was important to control mucosal inflammation. We further investigated the relationship between AMPK and metabolic pathways. It is well-established that AMPK is activated under conditions of ATP shortage in order to restore the decreased intracellular energy and thereby ensure the cell survival and maintenance of the cell function. After AMPK activation, ATP-consuming anabolic pathways are turned off, and ATP-producing catabolic processes are stimulated. Our results showed BBR decreased the total ATP content, suggesting that the increase in the AMPK activity was induced by BBR through a reduction in the ATP content.

BBR has been shown to inhibit OXPHOS, an oxygen-dependent ATP-producing pathway. OXPHOS is a highly efficient way of generating ATP, and almost all aerobic organisms carry out OXPHOS. BBR has been shown to inhibit OXPHOS^33,34^. We therefore further investigated the effects of BBR on the ATP production of colitis LPCD4^+^ T cells. In our study, as expected, BBR inhibited the OXPHOS of colitis LPCD4^+^ T cells. In our consecutive AMPK metabolic pathway experiments, we hypothesized that one of the anti-inflammatory mechanisms of BBR was the inhibition of OXPHOS, followed by ATP reduction, AMPK activation, and finally the reduction of inflammatory cytokines. Another study showed that AMPK activity was not involved in the reduction of inflammatory cytokines, such as IFN-γ and TNF-α, using WT-spleen CD4^+^ and CD8^+^ T cells in AMPK conditional knockout mice^15^; however, a precise analysis of the T cells infiltration in the immune mechanism-related IBD colon has not been performed. Thus, our data showing the mechanism of AMPK-related mucosal inflammation inhibition are novel findings and may highlight a new therapeutic target. The scavenging radical effect of BBR is likely to be a key mechanism underlying the anti-colitis effect, as the reduction of OCR and ATP was evident^35,36^. However, our present results indicated the strong effect of BBR on AMPK activation, and an AMPK agonist such as BBR may be expected to reduce inflammatory cell activation. We therefore emphasize the importance of AMPK in our immune-mediated colitis model as well as the effects of BBR on AMPK activation.

Our results also showed that BBR accelerated glycolysis. It is possible that this activation of glycolysis occurred as compensation for the loss of ATP by inhibiting OXPHOS. However, as we did not investigate the role of glycolysis in relation to LPCD4^+^T cells, the mechanism was not clearly defined. Previous reports have shown that inflammatory cells (e.g. effector memory T cells [T_EM_ cells]) tend to perform glycolysis. In contrast, anti-inflammatory cells, such as Foxp3^+^ regulatory T cells, more often perform OXPHOS^37^. Further studies are needed to gain more insight into the glycolysis of colitis LPCD4^+^T cells.

In our *in vivo* experiments, BBR increased the AMPK activity and suppressed inflammatory cytokines in colitic LP CD4^+^ T cells, suggesting that the same mechanism for suppressing inflammation as in *in vitro* experiments was involved. Concerning inflammatory cytokine control mechanisms by BBR, previous reports have shown that Bcl-2 and Foxp3^+^ Treg cells of LP CD4^+^ T cells were involved in regulating inflammation^38,39^. As previously reported, BBR reduced the expression of Bcl-2, suggesting the induction of apoptosis of inflammatory cells^30^. Given that our present model contained no natural Tregs, we could investigate only the induced Tregs^40^. The induced Tregs in our present model were increased by BBR administration, in line with the findings of a previous report^29^ (Fig. 5H,I). We also did not examine the detailed relationships between the effects of BBR and the characteristics of these cells, including AMPK activity, but the effects of BBR on these cells might offer further insight into the regulation of mucosal inflammation.

Our data obtained from *in vivo* experiments showed that BBR affected the gut microbiota. The gut microbiota have been postulated to induce IBD, including CD and UC^41^. The modulatory action of BBR was shown by changing the bacterial taxonomic structure and reducing the bacterial diversity. *Bacteroidetes*, a Gram-negative phylum, were significantly dominant under BBR supplementation, while the rate of *Firmicutes*, a Gram-positive phylum, was extremely low. A higher response to BBR of Gram-positive bacteria than of Gram-negative bacteria was reported in previous studies^42^. This phenomenon may be due to the permeability barrier of Gram-negative bacteria, which can eject both synthetic and natural toxins, including BBR alkaloids, through the activities of their multidrug resistance pumps. The penetration of this compound may also be limited due to the outer membranes of these bacteria^43,44^.

BBR had no effect on regulating the richness of gut microbiota in colitis mice, but this reagent seemed to cause a remarkable reduction in the gut microbiota diversity. The wide antimicrobial spectrum of BBR has been shown to decrease the diversity of intestinal flora^45,46^. Our data revealed that BBR reduced the severity of colitis and that the change in the gut microbiota was due to the anti-inflammatory effects of BBR. We therefore considered the change in the gut microbiota in our experiment to be reasonable.

In summary, we showed for the first time that BBR ameliorated CD4^+^ T cell-related chronic colitis in a mouse model with changes in gut microbiota via AMPK activity, possibly induced by the inhibition of OXPHOS, according to *in vitro* and *in vivo* experiments. The AMPK activity of colonic inflammation may represent a new therapeutic target for IBD, and medicines targeting the AMPK activity, such as BBR, may be recognized as new candidates for use in IBD therapy.

## Materials and Methods

### Animals

Balb/c and CB17-icr SCID mice were purchased from Japan CLEA (Tokyo, Japan). Mice were maintained under specific-pathogen-free conditions in the Animal Care Facility of Okayama University. The Balb/c donors and recipients were used at 5 to 7 weeks of age. For *in vivo* experiments of the BBR effect, donors and CB17-icr SCID recipients were used at 5 weeks of age.

All protocols and procedures conformed to the guidelines of the Okayama University Committee for Care and Use of Laboratory Animals and were approved by the Animal Experiments Ethics Committee of Okayama University.

### Chemicals

BBR (chloride form) was obtained from Sigma-Aldrich (St. Louis, MO, USA). The resulting compound used in this study had a purity of 98%. For *in vitro* experiments, BBR was added at a concentration of 100 µM. For *in vivo* experiments, we mixed normal diet with BBR at a concentration of 0.35%.

### Flow cytometry and antibodies of flow cytometry

To detect the expression of a variety of molecules on LP mononuclear cells, they were incubated with antibodies for 20 min. To detect the intracellular expression, after their surface molecules were stained, cells were fixed using a Cytofix/Cytoperm Kit (BD Pharmingen) and incubated with antibodies for 20 min. Multi-color flow cytometric analyses were performed using MACS (Miltenyi Biotec, Auburn, CA, USA), a Quant flow cytometer and Flowjo (FlowJo LLC, Ashland, OR, USA).

The following monoclonal antibodies (mAbs) were obtained from Biolegend (San Diego, CA, USA): APC anti-mouse CD4 (RM4-5), PE/Cy7 anti-mouse CD3 (145-2C11), PE anti-mouse CD25 (PC61), PE anti-mouse, IL-17A (TC11-18H10), FITC anti-mouse IFN-γ (XMG1.2) and Purified anti-mouse CD16/32 (93). The following mAbs were obtained from BD Pharmigen (San Diego, CA, USA): FITC anti-mouse CD45RB (16 A); PE anti-mouse Bcl-2 (3F11). The following mAbs were obtained from eBioscience (San Diego, CA, USA): FITC anti-mouse Foxp3 (FJK-16S).

### Induction of CD4^+^CD45RB^high^ T cell-transfer mouse colitis

CD4^+^ T cells were positivity isolated from the spleens of Balb/c mice using anti-CD4 (L3T4) (Miltenyi Biotec) according to the manufacturers’ instructions. Enriched CD4^+^ T cells were labeled with APC anti-mouse CD4 and FITC anti-mouse CD45RB. The CD4^+^CD45RB^high^ T cells were isolated using a FACS Aria I (Becton Dickinson). These cells were 98.0%–100% pure on a reanalysis. The isolated CD4^+^CD45RB^high^ T cells were intraperitoneally injected into the recipient CB17-icr SCID mice with 3 × 10^5^ cells per animal. After transfer, the recipient mice were monitored for clinical signs, including a hunched posture, piloerection, diarrhea, and blood in the stool. At autopsy, their clinical scores were assessed as the sum of three parameters^2^: hunching and wasting, 0 or 1; colon thickening, 0–3 (0, none; 1, mild; 2, moderate; or 3, extensive); and stool consistency, 0–3 (0, normal beaded stool; 1, soft stool; 2, diarrhea; 3, bloody stool).

*A histological examination* Tissue samples were fixed in phosphate-buffered saline containing 6% neutral-buffered formalin. Paraffin-embedded sections (5 μm) were stained with hematoxylin and eosin. Three tissue samples from the proximal and distal parts of the colon were prepared. The sections were analyzed without prior knowledge of the type of T cell reconstitution or treatment. The mean degree of inflammation in the colon was calculated using a modification of a previously described scoring system^2^, consisting of the sum of three parameters: crypt elongation, 0–3; mononuclear cell infiltration, 0–3; and frequency of crypt abscesses, 0–3.

### Isolation of mononuclear cells from murine organs

Single-cell suspensions were prepared from the LP as described^2^. To isolate LP CD4^+^ T cells, the entire length of the colon was opened longitudinally and cut into small pieces. The dissected mucosae were incubated for 45 min with Ca^2+^-, Mg^2+^- free Hanks’ balanced salt solution containing 1 mM dithiothreitol (Sigma-Aldrich) to remove mucus and then treated with 3.0 mg/ml collagenase (Roche Diagnostics GmbH, Berlin Germany) for 2 h. The cells were pelleted twice through a Ca^2+^-, Mg^2+^- free Hanks’ balanced salt solution and then subjected to Ficoll-Paque density gradient centrifugation. Enriched LP CD4^+^ T cells were obtained by positive selection using anti-CD4 (L3T4) MACS magnetic beads. Of the resultant cells, > 95% were CD4^+^ T cells, as determined by flow cytometry.

### Cell culture and treatments and intracellular staining of cytokines

LP CD4^+^ T cells from colitis SCID mice were cultured overnight with ionomycin (500 ng/ml), PMA (50 ng/ml) and BD GolgiStop (0.65 l/ml BD Pharmingen) mixed with 100 µM of sterile BBR^2^. After that, cells were collected, and their surface molecules were stained. Following cell fixation using a Cytofix/Cytoperm Kit (BD Pharmingen), the cells were incubated with PE anti-mouse IL-17A, FITC anti-mouse IFN-γ for 20 min. In some experiments, the designated concentrations (in text and figures) of Compound C (C.C.) and 5-Aminoimidazole-4-carboxamide ribonucleotide (AICAR) (Selleck Chemicals, Houston, TX, USA) were used to investigate the cellular functions of AMPK^15^. Each experiment contained a group with equal concentrations of DMSO as a control.

### Metabolic measurements

LP CD4^+^ T cells enriched from colitis SCID mice were cultured by the method mentioned previously. The extracellular acidification rate (ECAR) and oxygen consumption rate (OCR) were measured using with an xXF96 Extracellular Flux Analyzer under mitochondrial stress test conditions (Seahorse). Assay buffer was made from non-buffered RPMI 1640 medium supplemented with 2.5 mM dextrose, 2 mM glutamine and 1 mM sodium pyruvate (all from Sigma). The baseline ECAR and OCR values were averaged among at least two technical replicates per sample for the first three successive time intervals^47^.

### Measuring the intracellular ATP concentrations

LP CD4^+^ T cells enriched from colitis SCID mice were cultured by the method described previously. After that, cells were collected and plated at 5 × 10^5^ cells in 96-well plates. The cells were lysed with 100 µl lysis buffer and placed directly into the chamber of a luminometer (Flex Station3, Molecular Devices, CA, USA). Light emission was recorded after the addition of 100 µl of luciferin-luciferase solution (TOYO B-NET, Tokyo, Japan). The ATP values were averaged among at least two technical replicates per sample.

### Western blotting analyses

Total proteins were extracted from cultured LP CD4^+^ T cells using a protein extraction kit (Minute Protein Extraction Kit; Invent Biotechnologies, Inc., Eden Prairie, MN, USA). Extracted proteins were quantitated by the BCA method (BCA protein Assay Kit; Takara Bio, Inc., Otsu, Japan) and diluted to the same protein concentrations. Equivalent amounts of each group were run on SDS polyacrylamide gels. Proteins were transferred electrically to a PVDF membrane (Bio-Rad, Hercules, CA, USA). The membranes were blocked using polyvinylidene fluoride Blocking Reagent (Toyobo, Osaka, Japan) for 1 h. After that, the membranes were incubated with the following rabbit monoclonal antibodies overnight at 4 °C: anti-AMPK(Thr172), anti-stat1(Tyr701), anti-stat3(Tyr705), anti-JAK1(Tyr 1034/1035), anti-JAK2 (Tyr221) (Cell Signaling Technology, Inc., Beverly, MA, USA). After three washes with Tris-buffered saline with Tween-20 (Sigma), the membranes were incubated with secondary antibodies for 1 h at room temperature. The rabbit monoclonal antibodies were used at a 1:1000 dilution and the anti-rabbit secondary antibodies (Cell Signaling Technology) at a 1:2000 dilution. Bands were detected by using a LAS 4000 imager (GE Healthcare Life Sciences, Pittsburgh, PA, USA). β-actin was used as a loading control. Contrast was adjusted with Adobe Photoshop Elements 14. The quantity in each sample was analyzed using an Image J64 (NIH, Bethesda, MD, USA).

### Gut microbiome analyses of mice

Fresh fecal samples of mice were collected, immediately frozen by liquid nitrogen, and stored at −80 °C until use. The DNA purification of fecal samples was conducted according to the previous methods with slight modification^48^. The fecal DNA was sequenced using Illumina Miseq platforms according to the manufacturer’s recommendation (San Diego, CA, USA).

The raw sequences of both the BBR diet group and non-BBR diet group were processed using the QIIME software package, version 1.9.1 (http://qiime.org/) _ENREF_41^49^. After quality filtering, all sequences were normalized to the smallest number of reads (5324 reads) before being assigned operation taxonomic units (OTUs) using the Greengenes reference database with a 97% similarity threshold, and the alpha and beta diversity were compared. The relative abundance of OTUs in each group was evaluated at the phylum and genus levels. Chao1, observed species, Faith’s phylogenetic diversity whole tree and the Shannon diversity index were determined to estimate the alpha diversity. The weighted and unweighted UniFrac distance in beta diversity were evaluated to compare the microbial diversity between the BBR diet group and non-BBR diet group. All DNA sequences are available on the Sequence Read Archive under the relevant project identification number.

### Statistical analyses

Results are expressed as means ± standard error of the means for parametric data and medians for non-parametric data. All parametric data were compared using the Student’s t test, and non-parametric data were compared by the Mann Whitney U-test. Data were considered to be statistically significant at P < 0.05. The Statcel software program, version 3 (OMS Publishing Company, Saitama, Japan) was used for all statistical analyses.

## Supplementary information

Berberine improved experimental chronic colitis by regulating interferon-γ- and IL-17A-producing lamina propria CD4<sup>+</sup> T cells through AMPK activation

## Data Availability

Contact the authors or provide an SRA number.
