# Supplementary material for: Berberine improved experimental chronic colitis by regulating interferon-γ- and IL-17A-producing lamina propria CD4+ T cells through AMPK activation
Source: Sci Rep. 2019 Aug 15;9:11934. doi: 10.1038/s41598-019-48331-w (PMC6695484; doi:10.1038/s41598-019-48331-w)
Supplement: Supplementary file 1 — Berberine improved experimental chronic colitis by regulating interferon-γ- and IL-17A-producing lamina propria CD4+ T cells through AMPK activation [file 41598_2019_48331_MOESM1_ESM.docx]

**Berberine improved experimental chronic colitis by regulating interferon-γ- and IL-17A-producing lamina propria CD4^+^ T cells through AMPK activation**

*Masahiro Takahara^1^, Akinobu Takaki^1^, Sakiko Hiraoka^1^, Takuya Adachi^1^, Yasuyuki Shimomura^1^, Hiroshi Matsushita^1^, Tien Thi Thuy Nguyen^2,3^, Kazuko Koike^1^, Airi Ikeda^1^, Shiho Takashima^1^, Yasushi Yamasaki^1^, Toshihiro Inokuchi^1^, Hideaki Kinugasa^1^ Yusaku Sugihara^1^, Keita Harada^1^, Shingo Eikawa^4^, Hidetoshi Morita^2^, Heiichiro Udono^4^, Hiroyuki Okada^1^*

^1^ Department of Gastroenterology and Hepatology, Okayama University Graduate School of Medicine, Dentistry and Pharmaceutical Sciences, 2-5-1 Shikata-cho, Kita-ku, Okayama 700-8558, Japan.

^2^ Department of Animal Applied Microbiology, Okayama University Graduate School of Environmental and Life Science, 1-1-1 Tsushima-naka, Kita-ku, Okayama 700-8530, Japan.

^3^ College of Agriculture and Forestry, Hue University, 3 Le Loi, Hue City, Vietnam. ^4^Department of Immunology, Okayama University Graduate School of Medicine, Dentistry and Pharmaceutical Sciences, 2-5-1 Shikata-cho, Kita-ku, Okayama 700-8558, Japan. Corresponding authors: Masahiro Takahara, M.D., Ph.D., Department of Gastroenterology and Hepatology, Okayama University Graduate School of Medicine, Dentistry and Pharmaceutical Sciences, 2-5-1 Shikata-cho, Kita-ku, Okayama 700-8558, Japan. E-mail: mtakahara@cc.okayama-u.ac.jp

**Supplementary Figure**


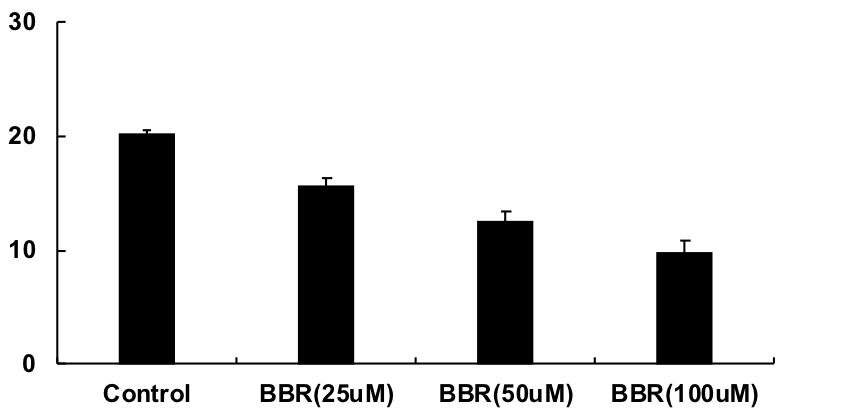


**% of IFN-γ^+^IL-17A^-^LPCD4^+^ T cells**

******

******

******


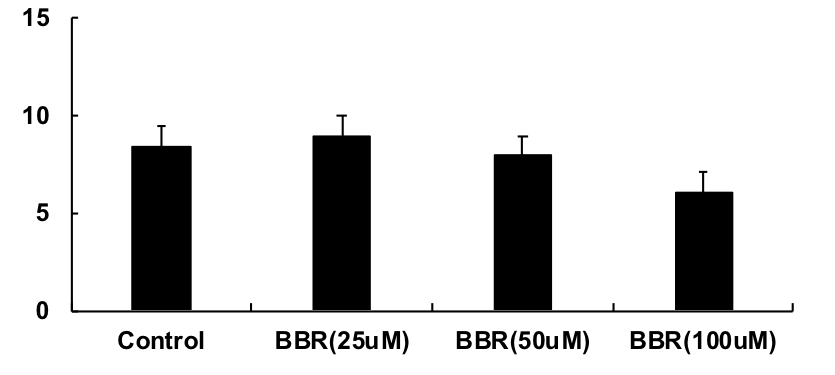


**% of IFN-γ^-^IL-17A^+^LPCD4^+^ T cells**

*****

**Supplementary Figure 1. (Related to Figure 1) The dose-dependent inhibitory effect of BBR.** The bar graphs show the proportions of IFN-γ- and IL-17A-producing cells in LP CD4^+^ T cells at the indicated BBR concentrations. *P<0.05, **P<0.01

**A**

**(130KD)**

**JAK1**

**(45KD)**

**β-actin**


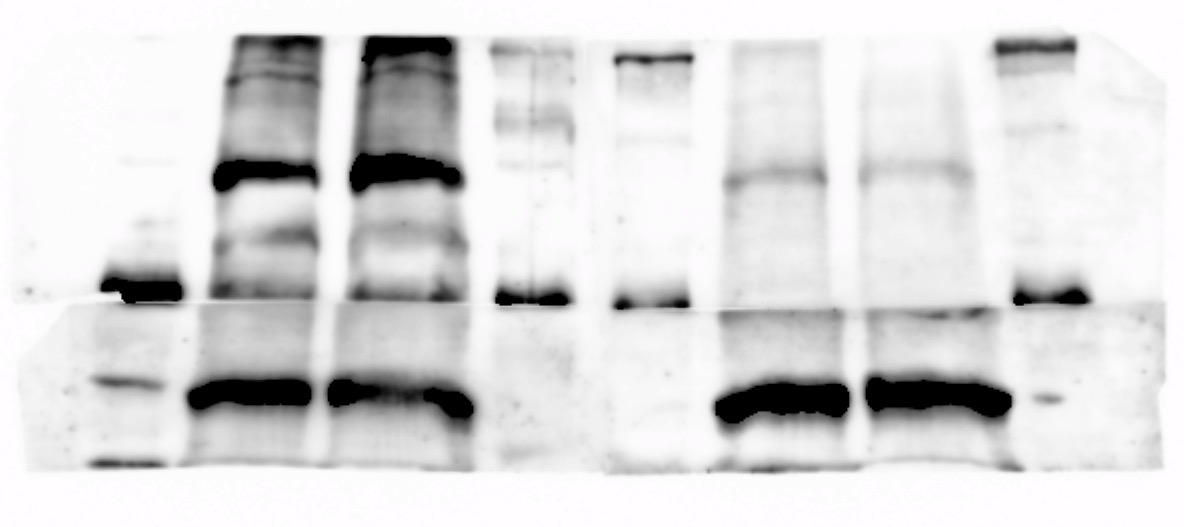


**C**

**B**

**C**

**B**

**(130KD)**

**pJAK1**

**
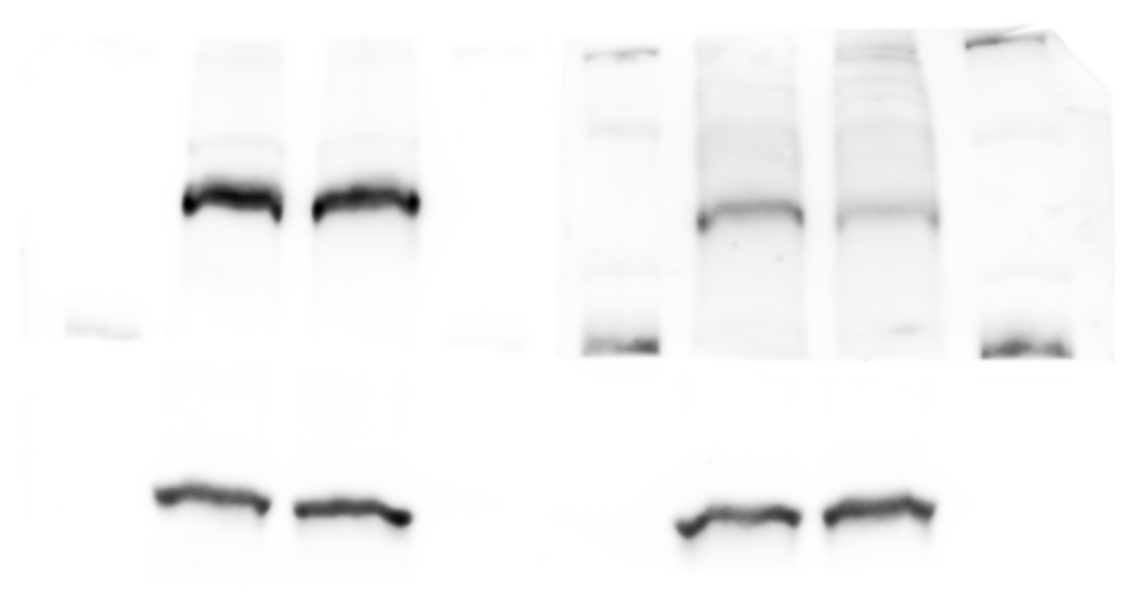
**

**C**

**B**

**C**

**B**

**B**

**(45KD)**

**β-actin**

**(125KD)**

**JAK2**

**(125KD)**

**pJAK2**

**C**


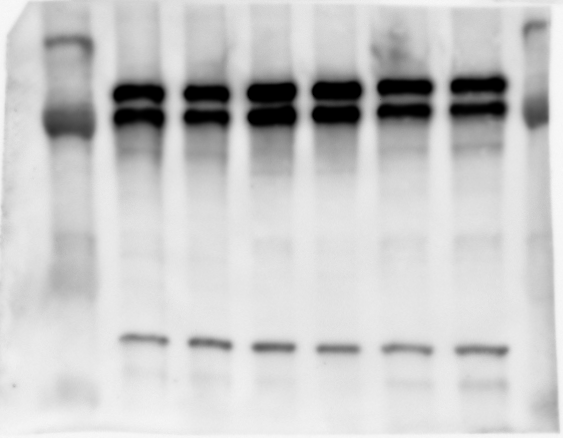


**Stat1**


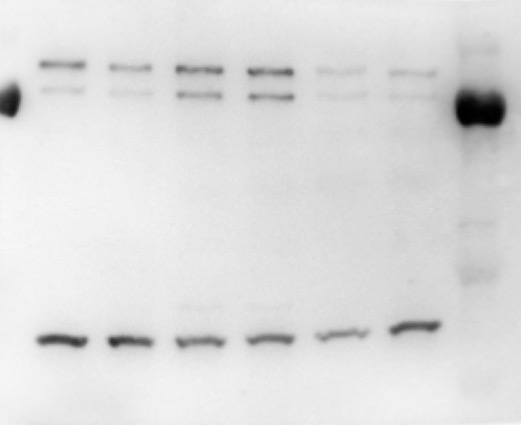


**(45KD)**

**(91KD)**

**Stat1**

**β-actin**

**pStat1**

**C**

**B**

**C**

**B**

**C**

**B**

**C**

**B**

**C**

**C**

**B**

**B**

**(84KD)**

**(45KD)**

**β-actin**

**Stat3**

**(79KD)**

**(86KD)**

**C**

**B**

**C**

**B**

**C**

**B**

**C**

**B**


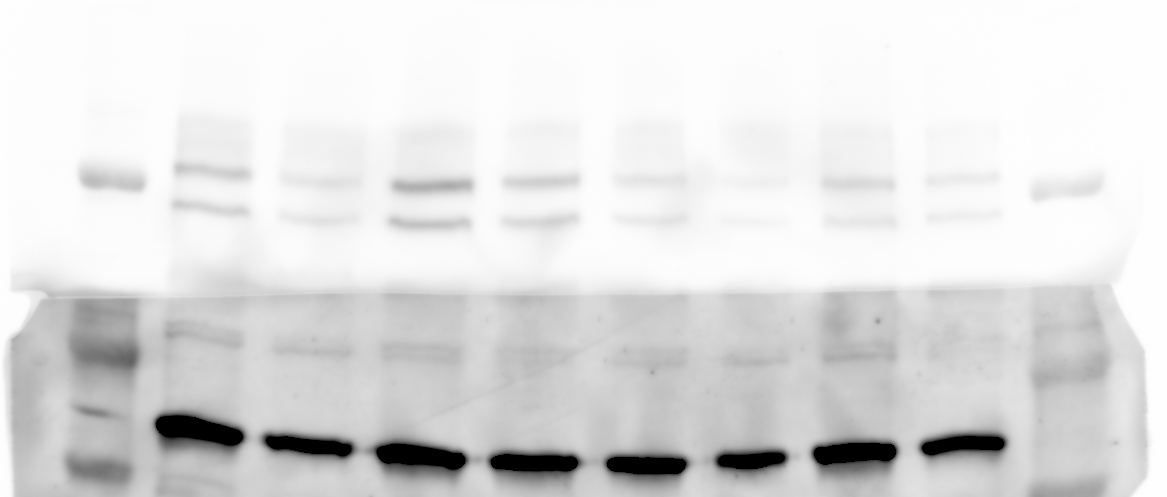


**C**

**B**

**C**

**B**

**C**

**B**

**C**

**B**


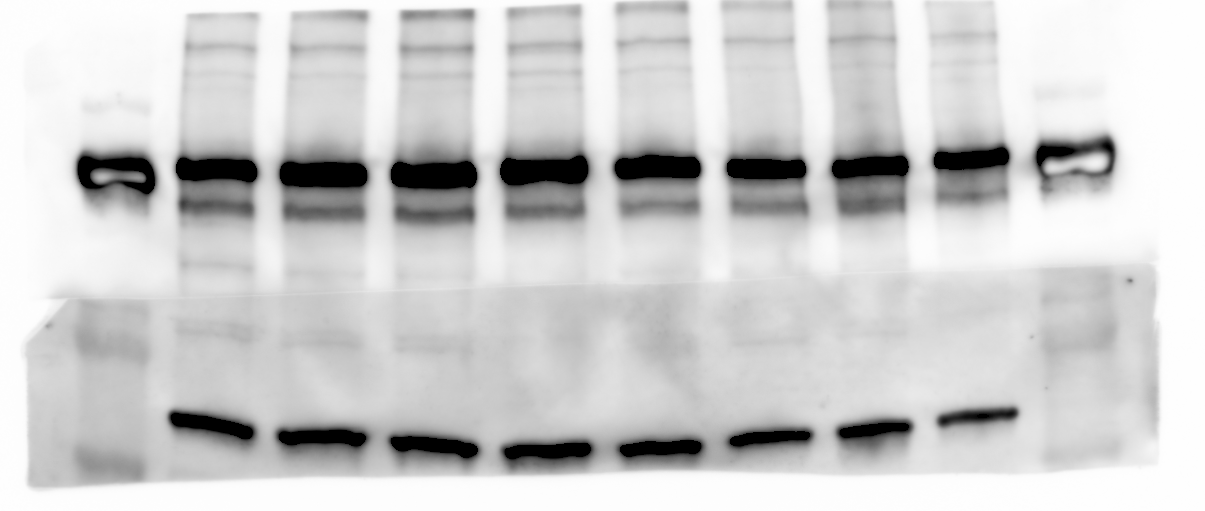


**D**

**Stat3**

**pStat3**

**Supplementary Figure 2. Full length blots of JAK1, JAK2, Stat1, Stat3 in Figure 2*.*** Red lines show the cropping locations. Blots showed representative one, three or

four examples. C: Control, B: BBR

**
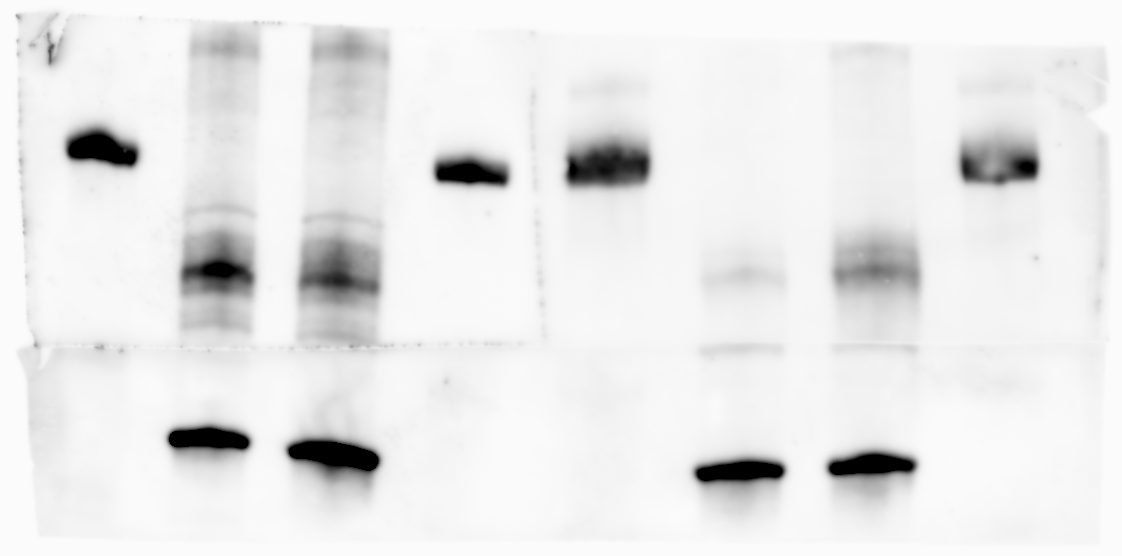
**

**A**

**C**

**B**

**C**

**B**

**(62KD)**

**pAMPK**

**(62KD)**

**AMPK**

**(45KD)**

**β-actin**

**D**

**A/C**

**A**

**C**


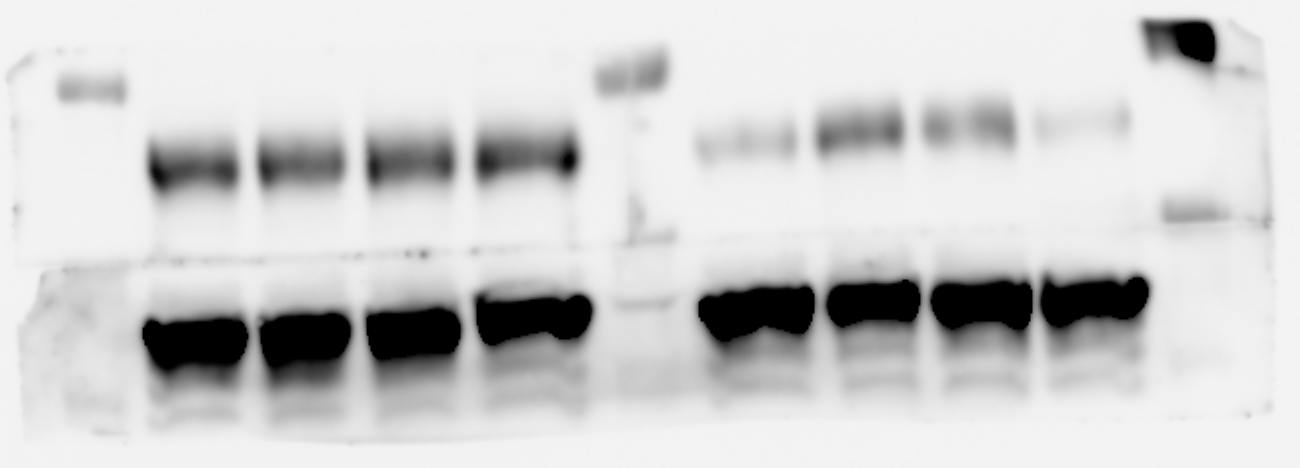


**D**

**A/C**

**A**

**C**

**B**

**(62KD)**

**pAMPK**

**(62KD)**

**AMPK**

**(45KD)**

**β-actin**

**Supplementary Figure 3. Full lengths blots of AMPK** **in Figure 3.** A,B: The red lines show the cropping locations. Blots show one example. C: Control, B: BBR, D: DMSO, A: AICAR, A/C: AICAR mixed with C.C, C: Compound C


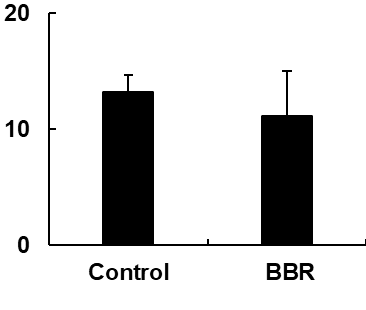


**Number (×10^6^)**

**N.S**

**Clinical Score**


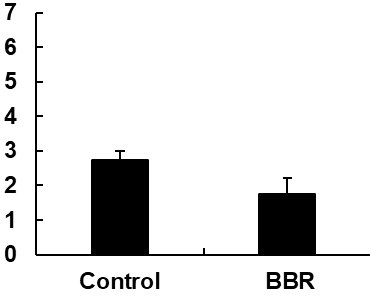


**N.S**

**B**

**A**

**C**


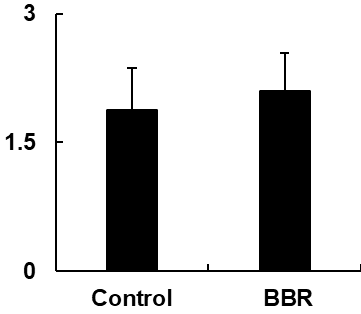


**% of IFN-γ^-^IL-17^+^**

**N.S**


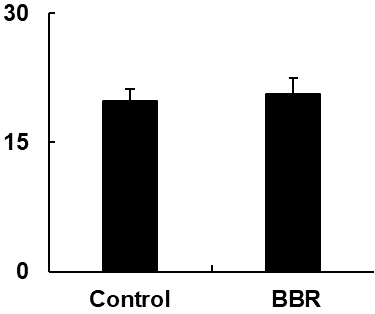


**% of IFN-γ^+^IL-17^-^**

**N.S**

**Supplementary Figure 4. (Related to Figure 5)** ***In vivo* experiments in animals fed a normal diet with BBR at a concentration of 0.12%.** A: Clinical scores, B: Number of LP CD4^+^CD3^+^ T cells, C: Proportions of IFN-γ- and IL-17A-producing cells in LP CD4^+^ T cells. N.S. not significant.

**(62KD)**

**AMPK**

**(45KD)**

**β-actin**


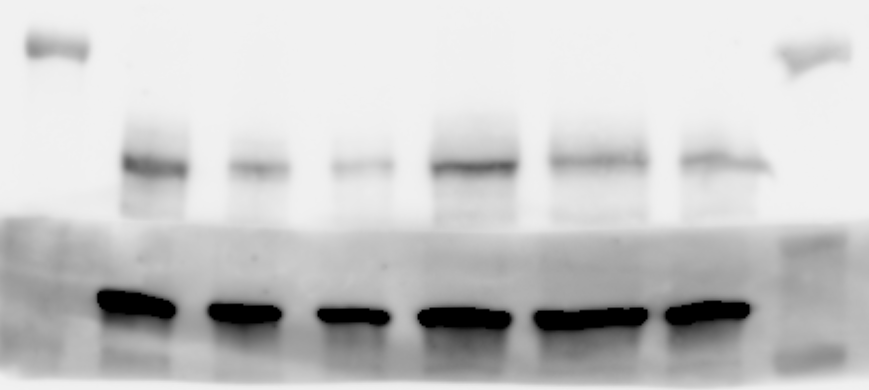


**pAMPK**

**C**

**C**

**C**

**B**

**B**

**B**


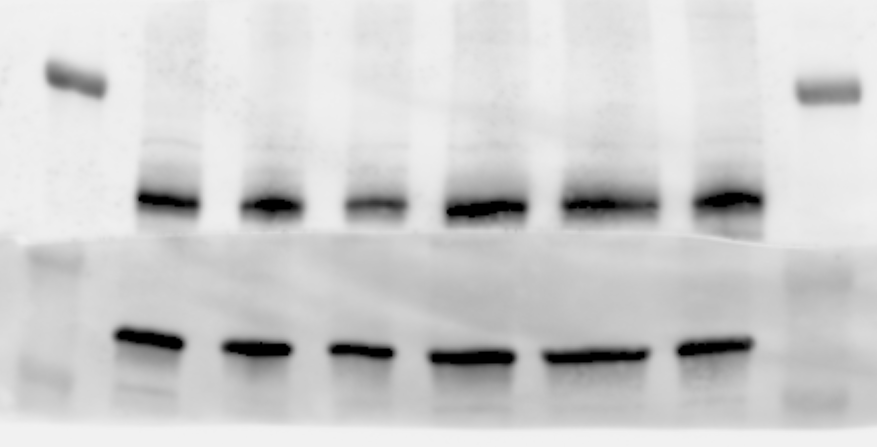


**AMPK**

**C**

**C**

**C**

**B**

**B**

**B**

**Supplementary Figure 5. Full length blots of AMPK in Figure 5.** Red lines show the cropping locations. Blots show three representative examples. C: Control, B: BBR.
